# The Euphrates Poplar Responses to Abiotic Stress and Its Unique Traits in Dry Regions of China (Xinjiang and Inner Mongolia): What Should We Know?

**DOI:** 10.3390/genes14122213

**Published:** 2023-12-14

**Authors:** Boniface Ndayambaza, Jianhua Si, Yanfang Deng, Bing Jia, Xiaohui He, Dongmeng Zhou, Chunlin Wang, Xinglin Zhu, Zijin Liu, Jie Qin, Boyang Wang, Xue Bai

**Affiliations:** 1 Key Laboratory of Ecohydrology of Inland River Basin, Northwest Institute of Eco-Environment and Resources, Chinese Academy of Sciences, Lanzhou 730000, China; ndayambazab78@mails.ucas.ac.cn (B.N.); jiab@lzb.ac.cn (B.J.); hexiaohui@nieer.ac.cn (X.H.); zhoudongmeng@nieer.ac.cn (D.Z.); wangchunlin@nieer.ac.cn (C.W.); zxinglin@yeah.net (X.Z.); liuzijin@nieer.ac.cn (Z.L.); qinjie18@lzb.ac.cn (J.Q.); wboyang@yeah.net (B.W.); bayxue2000@163.com (X.B.); 2 University of Chinese Academy of Sciences, Beijing 100049, China; 3 Qilian Mountain National Park Qinghai Provincial Administration, Xining 810000, China; dengyanfang23@yeah.net; 4 Faculty of Resources and Environment, Baotou Teachers’ College, Inner Mongolia University of Science and Technology, Baotou 014030, China

**Keywords:** desert regions, Euphrates poplar, gene family, abiotic stresses, transcription factors

## Abstract

At the moment, drought, salinity, and low-temperature stress are ubiquitous environmental issues. In arid regions including Xinjiang and Inner Mongolia and other areas worldwide, the area of tree plantations appears to be rising, triggering tree growth. Water is a vital resource in the agricultural systems of countries impacted by aridity and salinity. Worldwide efforts to reduce quantitative yield losses on *Populus euphratica* by adapting tree plant production to unfavorable environmental conditions have been made in response to the responsiveness of the increasing control of water stress. Although there has been much advancement in identifying the genes that resist abiotic stresses, little is known about how plants such as *P. euphratica* deal with numerous abiotic stresses. *P. euphratica* is a varied riparian plant that can tolerate drought, salinity, low temperatures, and climate change, and has a variety of water stress adaptability abilities. To conduct this review, we gathered all available information throughout the Web of Science, the Chinese National Knowledge Infrastructure, and the National Center for Biotechnology Information on the impact of abiotic stress on the molecular mechanism and evolution of gene families at the transcription level. The data demonstrated that *P. euphratica* might gradually adapt its stomatal aperture, photosynthesis, antioxidant activities, xylem architecture, and hydraulic conductivity to endure extreme drought and salt stress. Our analyses will give readers an understanding of how to manage a gene family in desert trees and the influence of abiotic stresses on the productivity of tree plants. They will also give readers the knowledge necessary to improve biotechnology-based tree plant stress tolerance for sustaining yield and quality trees in China’s arid regions.

## 1. Introduction

*P. euphratica* Oliv. (*P. euphratica*), the Euphrates poplar or desert poplar, is non-halophyte and mesophyte in its morphology, but has a high abiotic stress tolerance [1] and is an important component of riparian ecosystems in arid regions. This poplar tree species is mainly distributed in Southwestern Europe, including Spain [2]; Western Asian countries, including Iran [3], Iraq, Syria, and Turkey [4]; Central Asia, including Kazakhstan [5]; Pakistan [6]; India [7]; and China’s western Inner Mongolia, Xinjiang, and other arid regions [8,9,10,11,12]. It is also found in many other countries outside of Asia, such as the Middle East and North Africa (Kenya and Morocco [13,14]). *P. euphratica* is mostly found in the Chinese province Xinjiang, the Tarim Basin area, where it covers 89.1% of its total territory and is dispersed along rivers [15,16]. Although not a halophyte, it can adapt to extreme conditions, from flooding to extremely dry, hot atmospheres, with temperatures ranging from +54 °C to –45 °C, and from normal soil to soil with very high salinity (up to 2~5%) [17]. The adaptation of *P. euphratica* to high saline concentrations and high pH levels could be due to its physiological capacity [18,19,20]. Deserts have some of the harshest ecosystems because they combine high temperatures and low rainfall. Abiotic stress is an important variable affecting agricultural production and reducing tree yields [21,22]. Globally, these pressures include heavy metals, high pH, salinity, extreme temperatures (hot and cold), and extreme water levels (drought) [23]. The variety of plant evolution within gene regulatory profiles helps to control the water and ion balance, sustain proper photosynthesis, and resist abiotic stress [24,25]. These regulatory genes involve several physiological, metabolic, and cellular activities, including transcription, signal transduction, photosynthesis, energy metabolism, and protein synthesis and breakdown [26,27]. *P. euphratica* is a deciduous tree that may reach a height of 15 m and belongs to the Salicaceae family. This species is a dioecy, pollinated by the wind, and has different individuals for its male and female flowers [28]. Its fruits are capsules of tiny seeds produced in enormous amounts with cotton-like appendages [29], where these seeds disperse efficiently via wind at great distances. In phreatophytes, vegetative regeneration is very significant. *P. euphratica* roots spread virtually horizontally within the top 0.6 m of the soil, and the suckers they create can reach a distance of up to 40 m from their parent trees [30]. A phreatophyte found in arid areas is *P. euphratica* [31] because it can survive salt, which makes it an excellent natural genetic resource for developing plants with salt tolerance. *P. euphratica* has acquired a variety of morphological characteristics to cope with the salinity of its environment [32], including a unique hydraulic system [33] and succulent leaves [34,35]. Desertification occurs frequently in the riparian forests along the Xinjiang and Inner Mongolia sites, which are suitable for *P. euphratica* establishment [36,37]. To clarify the adaptation mechanisms that allow this species to live in the seasonal change-prone riparian ecosystem, *P. euphratica* uses the soil and trunk as water reservoirs to manage water stress during brief drought periods [38,39,40]. The restoration of many species in water-scarce places has a scientific foundation thanks to several studies, which also helps to improve the soil water balance and maintain the stability of the vegetation ecology in arid areas of China [41,42]. Consequently, countless studies confirm that gene families play a decisive role in the effects of drought, salt, and low-temperature stress on physiological and biochemical processes, which impede photosynthesis and destroy cell membranes.

**Figure 1:**
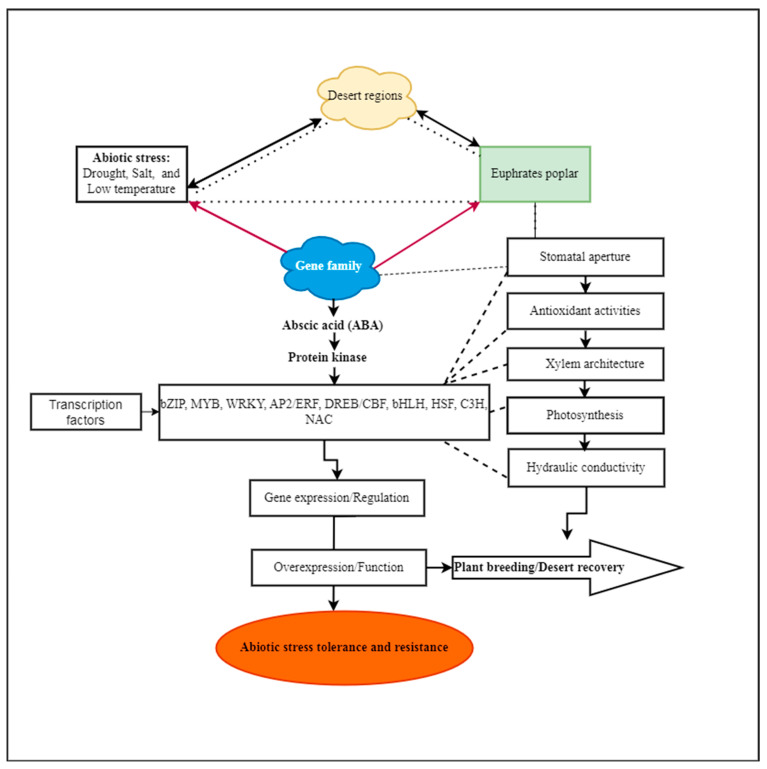
Abiotic stresses are perceived by *P. euphratica* throughout transcription regulation to ensure abiotic stress tolerance and resistance in arid areas.

In the last two decades, genomic information from decoded plant genomes, including *O. sativa* and *A. thaliana*, has employed DNA microarray technology with a bioinformatics method to evaluate the expression of genome-scale transcripts (mRNA) or transcriptomes. For instance, several miRNAs, including miR395, miR398, and miR399 in *P. tremula,* were enhanced under salt stress in microarray studies on forestry species. When *A. thaliana* was exposed to salt stress, miR398 was significantly down-regulated [43]. Under salinity conditions, *P. euphratica* displayed substantial changes in the expression levels of MiR168, miR1444, and miR1446 [44]. Under low-temperature stress, genes and their functions were analyzed [45]. With the increasing growth of omics data, bioinformatics methods have advanced [46], including those of genome, transcriptome [47], and proteome, alongside the quick development of computer technology [48]. Bioinformatics is also built on the fundamental idea that any biological mechanism comprises many molecular events, and that knowing the interplay within and between distinct levels of genomic architecture is the only way to comprehend phenotypic features [49,50]. Due to their high throughput and genome-wide performance, *P. trichocarpa* genome draft and poplar DNA microarray methods contribute to the inconclusive identification of gene functions [51,52,53,54,55]. Functional understanding, including metabolic pathways, protein complexes, and stress responses, is predicted through co-expression scrutiny. *P. euphratica* has also been studied [36,55,56]; the genome sequencing data were uploaded at the NCBI [56,57,58] and co-expressed the functions that respond to biotic stress. *P. euphratica* shows transcriptional modification in signaling, photoprotection, oxidative stress detoxification, and the suppression of stomatal closure, potentially changing drought stress responses (as illustrated in Figure 1) [47]. The expression of particular gene sets in plants determines their susceptibility to stress and their level of resistance to it. *P. euphratica* proteins’ functions in plant growth, development, metabolic pathways, and stress responses require advanced study for their regulatory roles and functions. Here, we gathered all information by using the Web of Science, the Chinese National Knowledge Infrastructure, and the National Center for Biotechnology Information. This review provides a quick summary of recent studies examining the functions of *P. euphratica* in novel gene families and molecular networks that may control plant tolerance to abiotic stress. These investigation have created the groundwork for improving the stress tolerance of forest breeding.

## 2. *P. euphratica* Gene Family Identification and Characterization

Environmental selection is the dynamic force that underscores molecular and biological evolution in *P. euphratica* [59]. The classification of plant tolerance may be based on its diversification, which includes obvious interspecific phenotypic and genomic changes [60]. As a result, due to the nature of plants, gene quantities, composition, and existence are all factors that are considered during the plant evolution process [61,62,63]. Importantly, this process of the evolutionary mechanism provides more information about the modifications in drought resistance, salt tolerance, and oxygen uptake as an outcome of the plant’s capability for adaptation during its life in the soil, such as the formation of the rooting system, vascular structure, and evolution of metabolites in response to abiotic stresses. Recently, a clear and profound understanding has been gained of *P. euphratica*’s genome analysis at the transcriptome level, which embodies conditional transcripts. The de novo assembly of *P. euphratica* reveals the most in-depth analysis of transcriptomic, gene annotation, profiling, gene expression, and transgenic lineages, providing an interesting concept for learning about their gene family. Therefore, the most prevalent TFs corresponding to the bZIP, bHLH, C3H, and NAC TF families were those differentially expressed in the MYB and MYB-related transcription factor families [47]. For example, some genes are crucial in transport, cellular communication, and metabolism. A gene family helps us to identify and characterize the shared origin of proteins with related structures and metabolic activities. Based on the *P. euphratica* resistance gene family, it is evident that this model plant is tolerant not only to external stress, but also to the multiple environmental pressures during adaption. For example, the transcription factors (TF gene family) function as the regulators of *P. euphratica*, including plant growth, developmental, and reproductive functions. Studies have established that progress in *P. euphratica* can be realized based on the different numbers of resistance genes, comprising the *PeDREB2* gene family [64]. For example, downstream of the DREB2A transcription cascade system, where *PeDREB2*, *PeCPK10*, and HsfA3 are expressed in drought and salt stress [65]; they were also induced by cold, but not by abscisic acid (ABA) treatment [64,65,66]. It is interesting that these gene families play particular roles in tree plants, such as molecular chaperones (heat shock proteins). This large family is a global salvation system in living organisms that prevents protein damage by stopping aggregation, refolding, and restoring cellular homeostasis through proteolysis and lysosomes/proteasomes. Additionally, some extremely hydrophilic proteins, such as the LEA and COR family members, may serve as chaperones to protect proteins and membranes from damage caused by stress [67].

Under abiotic stressors, the gene family contributes significantly more at the transcription level (Figure 1). For example, heat, drought, and salt stresses consequently significantly elevated the expression of *PeuHsf* genes in *P. euphratica* [68], as well as bHLH (*PebHLH35* from *P. euphratica* offers drought resistance by controlling the growth, photosynthesis, and stomatal development of *A. thaliana*) [69]; R2R3-MYB [70]; WRKY [71]; WRKY1 (because *PeWRKY1* was able to connect to the W-box in the *PeHA1* promoter, salt stress increased the transcription of *PeHA1*) [72]; *PeuSAP* [73]; GASA [74]; TCP [75]; and the SPX gene family, which plays a role in the response mechanism to phosphorus stress [76]. It has been witnessed that the transcription factors of the large family play a crucial role in the *P. euphratica* species [47]. Data from high-throughput sequencing revealed that *P. euphratica* NAC (*PeNAC*) genes play a role in osmotic and salt stress responses. One study showed that distinct stress-responsive *PeNAC* transcription factors differentially control salt tolerance in transgenic plants [77]. For example, the stress response gene *PeNAC1* in *P. euphratica* was subjected to salt stress; despite that, transgenic *A. thaliana* that overexpressed *PeNAC1* had greater salt tolerance. The cloning and functional analysis of PeNAC045 also demonstrated high responses to abiotic stress [78]. The successful identification and isolation of the strongest genes have been experimented with, which suggests the crucial derivative stress tolerance of the gene family in *P. euphratica* and other model plants. In addition, the cloning gene has evidenced the critical role of *P. euphratica* tolerance to drought stress, including the *PeSCL7* gene [79]; *PeSTZ1*, a C2H2-type zinc finger enhanced the freezing tolerance throughout the modulation of ROS regulation within the *PeAPX2* expression [80]. Increasing the water transport capacity in *P. euphratica* LAC2 (LACCASE) increases its drought tolerance [81]. Importantly, the overexpression of *PeuLAC2* in poplar plants alters the xylem structure, thickening the secondary cell walls and increasing the fiber cell length and stem tensile strength. *Arabidopsis* CBF gene overexpression induces orthologs and increases stress tolerance. In transgenic poplar leaves, drought, high salt, and cold stressors increase *PeCBF4a* expression, which enhances the photosynthetic capacity and PSII photosynthetic electron transport activity.

Additionally, most physiological mechanisms depend heavily on the photosynthetic activities of *P. euphratica.* For example, exogenous plant growth factors are essential for *P. euphratica*’s ability to withstand drought by controlling photosynthesis and lowering salt stress [82]. Additionally, *P. euphratica* demonstrates heat tolerance and leaf protein accumulation [83]. What about the modifications made in reaction to salinity in the roots of *P. euphratica*, then? For instance, to respond to this query, extracellular factors, such as calmodulin, calmodulin-like, and calcineurin B-like proteins, encourage the production of calcium. The Ca^2+^-mediated CBL-CIPK network, which affects salt tolerance, depends on CIPKs [84,85]. It has been well established that several CIPK genes are implicated in the SOS signaling pathway that mediates salt tolerance [86]. The Na^+^/H^+^ antiporter controls Na^+^ efflux and root salt tolerance in *A. thaliana* [87,88], while the interaction between *AtCBL4* and CIPK24 (SOS3-SOS2 complex) mostly controls NHX activity [87] as opposed to the shoots, where the *AtCBL10*-*AtCIPK24* complex defends against salt stress [86,89]. *PeCBL1* interacts with CIPK24, CIPK25, and CIPK26 to improve *P. euphratica*’s ion homeostasis and salt tolerance. These CDPKs are substrates for ion channel proteins and transporters, and are implicated in abiotic stress-induced Ca^2+^ signaling [90,91]. For instance, *PeCIPK26* expression was found in the roots, stem, leaf, cell membrane, cytoplasm, and nucleus, possibly induced by salt stress [92]. By overexpressing the *PeCIPK26* gene in *A. thaliana* cipk24 mutants, the role of *PeCIPK26* in salt tolerance was examined. The faster germination rate, lower Na(+) buildup, and greater capacity to release Na(+) when grown with NaCl of transgenic plants compared to mutants demonstrate their superior salt tolerance. These findings point to a role for *PeCIPK26* in *P. euphratica*’s response to salt stress. The model plant regulates genes involved in drought, salt, and freezing stress, indicating resistance to these environmental conditions. For tree-breeding programs, these genes work as abiotic stress markers and aid comparative genomics projects, revealing shared and distinctive transcriptional pathways between tree species [70]. This poplar species revealed its transcriptional pathways that lead to the discovery of the variance in expression of H^+^-ATPase, rubisco activase, laccases, and expans in *P. trichocarpa*, but not in *P. euphratica* [93,94]. *A. thaliana*’s reactions to drought show clear species-dependent characteristics [95]. This could indicate that angiosperm trees, independent of species or hybrids, will likely have various applications in response to drought stress, and that these processes are extremely varied among species and genotypes. Comparing tree transcriptome investigations’ outcomes is challenging due to varying responses based on plant organs, developmental stages, and stress levels [96]. In *P. euphratica*, extreme stress triggers quick reactions, while progressive water depletion enables plants to adjust by putting stress defense mechanisms in place. The intricacy of gene expression studies in response to abiotic stress is shown by the fact that various water depletion regimes activate various gene networks (as illustrated in Table 1). Identifying new genes for salt and drought tolerance in plants can advance tree breeding and ecosystem restoration in woody plants.

## 3. Effect of Salt Stress in *P. euphratica*

**Figure 2:**
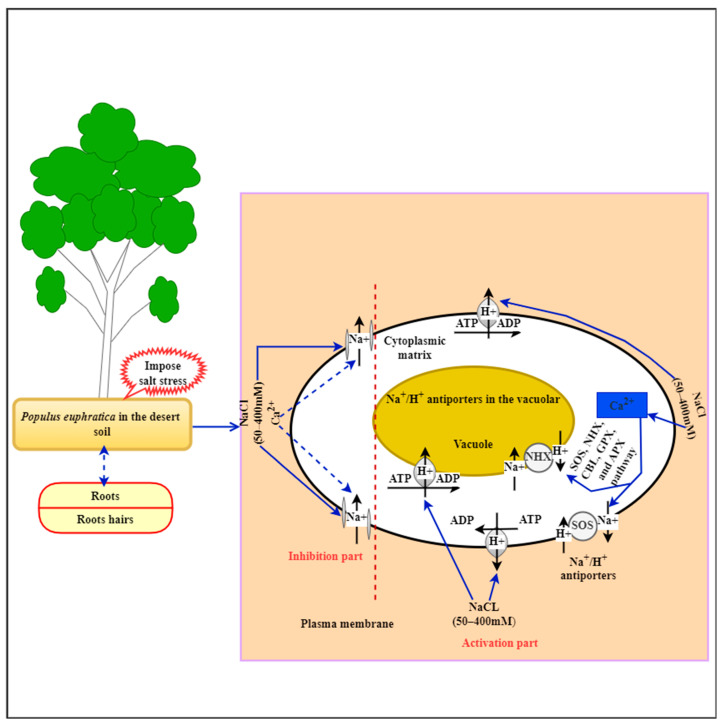
A proposed diagram model of Na^+^/H^+^ homeostasis in *P. euphratica* during NaCl stress tolerance in arid areas.

Around 60% of the world’s land surface is exaggerated by salt, which spreads due to ineffective irrigation practices or water contaminated by salt. The total amount of soluble salts in the soil is measured as soil salinity, and high levels can kill or induce plant wilting. Salts, including NaCl, CaCl_2_, gypsum, magnesium sulfate, potassium chloride, and sodium sulfate, are frequently found in saline soils. As a result of salt growth in the cytoplasm, high salinity can harm and even kill leaves. Halophytes are salt-tolerant plants that can survive in environments with salinities higher than 400 mM. Terrestrial vascular plants depend on xylem water transfer and stomatal evaporation to survive in stressful environments. The leaves of *P. euphratica* are wax-coated, rigid, and thick, preventing oxidation-related damage. They grow more robust xylems with drought-induced cavitation due to their adaptation to hydraulic conductivity and embolism. *P. euphratica* grows in dry, hot climes; increases its photosynthetic rate and evaporation; accumulates salts; deepens its tape roots; and increases its salt content. The primary root penetrates one meter of soil vertically, while the lower end develops lateral feeder roots. However, salt stress has a major negative impact on plant development, growth, and reproduction. Understanding salt tolerance pathways is essential, as rapid exposure activates genes involved in ribosome activities, photosynthesis, cell development, and transport. Reactive oxygen species (ROS) in excess can harm organisms by degrading chlorophyll and causing membrane leakage. It is necessary to find tree and woody plant species that can withstand salt and improve their resistance. The *P. euphratica* tree is a good example of a tree with salt tolerance. *P. euphratica* has lengthy juvenile periods and frequently reproduces across several years. One of the main characteristics that sets *P. euphratica* apart from other plants is its secondary growth. It has the capacity to produce thickened vascular bundles that accumulate to create secondary xylem (dicots) or wood-like tissue (monocots), which allows them to improve their transport capacity when needed. This species has high outcrossing rates, long-distance pollen distribution, large effective populations, an arborescent stature, longevity, and late successional communities. These characteristics could make *P. euphratica* less susceptible to genetic bottlenecks and more resilient to habitat fragmentation and climatic changes. Tissue-specific differentially expressed genes (DEG) under salt stress has diverse functions, with membrane transporter activity being the most significant leaf function and the oxidation–reduction process being the most significant root function. Gene families like SOS, NHX, GolS, GPX, APX, RBHF, and CBL are involved in ionic homeostasis in *P. euphratica* seedling tissues [99]. DEGs, such as antioxidant genes, contribute to ROS scavenging and plant salinity tolerance by maintaining ionic and ROS homeostasis in tissues and improving ion uptake, transport, and compartmentalization [100,101]. The regulation of pathways, including plasma membrane and tonoplast Na^+^/H^+^ transporters, is crucial for salt stress tolerance. *P. euphratica* halophytes maintain a low Na^+^ influx and prevent Na^+^ accumulation [102,103]. Ionic homeostasis is accomplished through genes that code for pyrophosphatase, cation/proton antiporters, plasma membrane and vacuolar H^+^-ATPases, and salt tolerance systems (as illustrated in Figure 2) [104,105]. *P. euphratica* studies reveal genes controlling the cells’ salt tolerance, ion compartmentalization, xylem loading, and potassium levels [18,106,107]. *P. euphratica* has intricate and interrelated defense mechanisms that help it avoid or lessen environmental harm. As part of these systems, transcription factors (TFs) bind to cis-elements in the promoters of target genes or other useful modular structures to regulate how genes are expressed in response to abiotic stress. In *P. euphratica*, 2382 TFs (2382 loci) have been discovered and categorized into 58 families following the family assignment guidelines. The *P. euphratica* genome has a high content of TFs (http://planttfdb.gao-lab.org/index.php?sp=Peu, accessed on 6 July 2023) and various transcription factors, including DREB, bZIP, AP2/ERF, WRKY, and bHLH, that regulate plant responses to stress (as mentioned in Figure 1), including salt stress [108,109]. In non-woody plants, WRKY genes have just been discovered; nonetheless, the effects of salinity on these transcription factors in woody plants may be pertinent. Interestingly, researchers have revealed that salt stress inhibits the *PalWRKY77* gene by reducing the salt tolerance in *P alba* var. *pyramidalis* [110]. The *PalWRKY77* pathway receives a bad signal from this ABA regulatory mechanism, making poplar trees more vulnerable to salinity. However, the salt-induced transcriptional response of *PeWRKY1* in *P. euphratica* reveals that WRKY1 binds to H^+^-ATPase promoters, improving gene expression and salt tolerance [72]. However, little is understood about the modifications that the *P. euphratica* xylem undergoes in response to salinity. Addressing the other transcription factor members is needed to facilitate a molecular revolution for tree breeding in other species.

## 4. Effect of Drought Stress on the Physiology of *P. euphratica*

According to different studies, typical abiotic stress, known as drought, impacts cellular homeostasis, gas exchange, seed generation, plant development, and water relations [111,112]. Plants have evolved innate defenses against drought stress to adapt to harsh settings, such as closing stomata, decreasing transpiration, producing abscisic acid (ABA), and storing hydrogen peroxide (H_2_O_2_) [113]. Although drought can severely restrict a plant’s ability to grow and develop, family genes are thought to have a key role in how the plant reacts to various conditions. Drought impacts the physiological environment of the soil microbiota and plants. Auxins, cytokinins, gibberellins, and abscisic acid, among other phytohormones, are produced by bacteria and have been shown to increase drought resilience in *P. euphratica* [114,115]. As plant-growth factors, due to their capacity to produce endospores, which enable bacterial survival for lengthy periods under unfavorable environmental conditions, bacterium genera, including *Bacillus* sp., are frequently discovered in arid land [116]. At the same time, the various metabolisms and strong physical tolerance of *Pseudomonas* sp. isolates in *P.euphratica* cultivated in desolate and saline soil may explain their large abundance [117]. Plant rhizobacteria support plant development, biological regulation, and resistance to abiotic stress through direct and indirect processes [118]. The direct mechanisms involve phytohormone regulation, the release of volatile compounds, and an enhanced plant uptake of nutrients [119]. The indirectly beneficial effects include suppressing deleterious microorganisms and pathogens, competition for nutrients, inhibiting enzymes, and triggering host-induced systemic resistance [120,121]. The rhizosphere of both sexes suggests the presence of sex-specific variation in bacterial communities and their relative abundances [122]. In reaction to dryness, males have more drought-tolerant fungus and bacteria in their rhizospheres than females [123]. The altered bacterial and fungal community composition increase soil ammonification in the rhizosphere of female plants. The contribution of Rhizobium in biocontrol activities against pathogens and the alleviation of stresses play a decisive role in the *P. euphratica*. For instance, recent discoveries discussed the significance of rhizobia, which promote plant growth by reducing salt and osmotic stressors in contaminated soils (see [124] and references therein).

Additionally, rhizobium occurrence and utilities in microbiomes of non-leguminous plants were reviewed to control the growth of various soilborne plant pathogens by focusing on the biological control of the different genera [125]. *Rhizobium populi* sp. nov., an endophytic bacterium recovered from *P. euphratica*, was particularly isolated from the storage fluids in the stems of *P. euphratica* trees, which is an interesting development [126]. The studies conducted on the pathogenic fungus in the pathogenic site of *P. euphratica* found that it increased its survival rate in arid regions [127]. Microbiome research indicates that planting *P. euphratica* may influence bacterial communities, possibly resulting in more infections. However, research on the relationship between pathogenic bacteria and a high rate of plant mortality is lacking. Different research revealed that the synergistic actions of the dioecious *P. euphratica* roots and coexisting microorganisms allow them to respond to and survive drought stress [128,129]. However, research on the identification of genes associated with microorganisms to cope with abiotic stresses remains scarce in this model tree plant. *P. euphratica* grows in deep water, relying on water table depth, but faces drought-induced cavitation, causing shoot cessation, stomatal closure, and reduced root growth [130,131]. Recent findings have also unveiled that long-term irrigation in *P. euphratica* plantations affects soil phosphorus fractions and microbial communities [132]. They found positive relationships between inorganic P and various bacteria, while negative associations were observed with Burkholderiaceae and soil phosphorus (soil P) in its inorganic form (Pi). The study suggests that water management techniques focusing on soil microbial recovery could improve soil quality. However, the increased mineralization of organic P in *P. euphratica* is linked to soil moisture, pH, and microorganism profiles, necessitating future research on foliar P fraction distribution. Based on the research conducted on the impact of cow dung and biochar on phosphorus efficiency in *P. euphratica* soil, bacterial communities, and functional genes (phoC, phoD, gcd, and pqqC; see [133] and references therein), the authors found that returning cow dung improves soil properties, seedling growth, and phosphorus availability, which are up-taken throughout the roots of *P. euphratica*. Biochar, a carbonized form of cow manure, has a more definite cumulative phosphorus content and promotion of bacterial diversity in arid regions. However, the study suggests increasing biochar use in plantation management and conducting a long-term analysis to discover the utmost scientific addition technique for *P. euphratica* seedlings and cow dung. In desert environments, mycorrhizal associations and symbiotic interactions between fungi and plant roots increase plant resilience to drought stress [134,135]. These connections aid in the intake of nutrients, particularly phosphorus, which is necessary for plant growth [136]. These plants survive in arid conditions by developing specialized mycorrhizal associations, which enhance the soil’s surface area for the uptake of nutrients and water, as well as delivering carbon compounds from the host plant [137]. It is interesting to note that *P. euphratica* may also help us to fully comprehend the intricate mechanisms underpinning *P. euphratica*’s resilience to drought stress and salt stress, which are mediated via mycorrhizal connections in the desert. The study reveals that not all arbuscular mycorrhizal fungi (AMF) can infect and colonize plant roots. In extreme conditions, one species can become predominant. The alkaline *P. euphratica* rhizosphere soil favored *G. mosseae* growth, suggesting its selectivity and adaptability. Other AMF species may survive in the rhizosphere soil or colonize *P. euphratica* roots. There is, however, a small gap in this study that has to be filled [138].

Photosynthesis is one the most significant processes in a plant’s life, but drought stress may affect its mechanisms, which stops plant growth and development under severe ecological conditions. The direct impacts of drought stress on photosynthesis include fluctuations in photosynthetic metabolism and constraints on diffusion via the stomata and mesophyll. Secondary effects include oxidative damage brought on by the superposition of various stresses. Interestingly, a thorough investigation comparing salt and drought stress found that both conditions resulted in the down-regulation of several photosynthetic genes. In *P. euphratica*, for example, the roots and leaves suggested that during stress, protein concentration can be altered without affecting gene expression [131,139]. Importantly, at the transcriptome level, 27 photosynthesis genes were differentially expressed during drought stress, with the majority being down-regulated and six genes enhancing expression [47]. For examining the processes of abiotic tolerance in woody plants, *Populus euphratica* is a potential candidate species. For instance, under salt and drought stress conditions, *PeGSTU58* overexpression lines showed increased expression of various stress-responsive genes, such as DREB2A, COR47, RD22, CYP8D11, and SOD1. Additionally, *PebHLH35* has been demonstrated to be able to directly bind to the promoter region of *PeGSTU58* and stimulate its expression in yeast one-hybrid experiments and luciferase studies. These findings suggested that *PeGSTU58*, whose expression was favorably controlled by *PebHLH35*, had a role in the tolerance to salt and drought stress by maintaining ROS homeostasis [140].

## 5. Mechanism of Abiotic Stress Tolerance in *P. euphratica*

Developing mechanisms that allow plants to regulate their water loss while continuing to fix carbon dioxide (CO_2_) through photosynthesis has been a critical step in plants’ colonization of terrestrial environments [141,142]. This is an important step, since, in a natural environment, water availability is undoubtedly the primary determinant of plant distribution and survival. Drought tolerance mechanisms are any processes that let plants continue to grow or produce under conditions of an insufficient soil water supply. The first tactic is to prevent a water deficit. Drought resistance entails finishing the life cycle under ideal conditions, limiting transpiration, and maximizing root uptake [143,144,145]. It also involves increasing deciduous plants that hibernate during droughts, as well as species of arid environments with permanent access to the water table, like *P. euphratica*. By sacrificing biomass production, these strategies enable plant survival. One of the main issues restricting most plants’ survival ability is water. Plants, particularly those found in arid regions, have developed a variety of tactics to stop water loss or adapt to growth in water-scarce environments. At the physiological level, for instance, *P. euphratica* demonstrates greater antioxidant enzyme activity and stronger root hydrotropic development than other poplar plants [146]. The capacity of stems to carry water (called xylem pressure) and the closure or opening of stomata controlled by phytohormones support water balance in plants (Figure 1). Numerous studies on woody plants have demonstrated that drought tolerance is closely tied to xylem structure, which is connected to the ability of plants to transport water [147,148,149,150]. The experimental evidence, however, is in favor of the associated evolutionary processes, particularly when it comes to the molecular control mechanisms of *P. euphratica*. It offers a variety of ecological services as a natural check on the spread of deserts, including preventing sandstorms, controlling oasis conditions, and even preserving the ecosystem balance. *P. euphratica* is therefore frequently used as a model woody plant for the investigation of trees’ abiotic resistance mechanisms. *P. euphratica* has developed a variety of adaptations in arid environments. Of these, the polymorphisms in its leaves and its hard wood are thought to be two crucial adaptive features that may give *P. euphratica* an enhanced adaptability to desert conditions. For instance, the xylem of *P. euphratica* wood can store significant amounts of cellulose and lignin [151]. The xylem’s secondary cell wall (SCW) serves as a route for the long-distance movement of nutrients and water, supporting plants mechanically and as a barrier against disease and insect attacks [152,153,154]. These characteristics and leaf polymorphisms enable the plants to show physiological responses that aid their adaptation to severely dry settings, such as reduced photosynthetic activity, altered cell wall flexibility, and stomatal aperture regulation.

Moreover, the ultimate impact of abiotic stress on plant growth depends not only on the severity of the damage but also on how well the plant can recover from the damage [155]. Photosynthetic electron transport and carbon metabolism rates often decline less in plants that can withstand cold temperatures [156]. Compared to cold-sensitive genotypes, these modifications let these plants recover more quickly from chilling stress [157].

## 6. *P. euphratica*, a Naturally Stress-Tolerant Tree, as a Source for Finding Stress-Adapted Genes

*P. euphratica* is the only arbor species among the toughest plants that can live in the deserts of the Inner Mongolia Autonomous Region in north-central China [158]. Therefore, the Taklamakan Desert in Xinjiang is the second-largest sand desert with moving dunes and is known for its harsh conditions [159]. The Tarim River, the longest inland river in China, flows through the desert, fed by melting snow from Tianshan Mountains [160]. Ejina Banner in Inner Mongolia has had a *P. euphratica* tree growing there for over 800 years [2,161,162]. The locals refer to it as a “sacred tree.” A new plant species appeared along the ancient Mediterranean Sea some 65 million years ago, following the extinction of the dinosaurs [163,164]. Its scientific name is *P. euphratica* and its binomial name is *P. euphratica* Oliv.

The leaves are formed like willow leaves as a sapling to lessen evaporation. Its leaves enlarge and take on a rounded form as it becomes taller and requires more energy to sustain the trunk. In the Gobi Desert, the poplar thrives on water but is drought-resistant. The poplars can flourish happily as long as the groundwater stays at a depth of roughly four meters below the surface. An adult *P. euphratica* tree can release ten kilograms of salt and alkali annually. Water and soil are well preserved in the desert with a poplar forest. The Taklamakan Desert demonstrated that it has phreatophytic and deciduous character. Despite having a low drought tolerance, the ongoing contact with groundwater allows for significant water usage and transpiration through the mounting season. This method of avoiding drought makes accumulating a lot of biomass possible. Studies have shown that *P. euphratica* is a fast-growing tree species but is stress-sensitive, and our focus has increased due to its use in environmental protection, agroforestry on marginal soils, and afforestation on damaged soils [18,165]. The study of the endophytic bacteria of *P. euphratica* in various saline–alkaline regions of Xinjiang revealed that the bacteria in the various tissues of *P. euphratica* changed with the change in soil salinity [166]. Endogenous bacteria diversity is lower in *P. euphratica* sap tissue under high salinity. These unique bacteria under various salinities were primarily related to the host’s stress tolerance [20]. Understanding the connections between natural microorganisms and stress-tolerant trees can enhance plant performance and broaden the variety of tree plants. In climate change situations, integrated genomic, transcriptomic, proteomic, and metabolomics data can reveal mechanisms for stress tolerance [167,168]. Creating DNA, RNA, protein, and metabolite separation procedures for woody plant biology and natively tolerant tree biology, including the recalcitrance of seeds, viability, and seed germination and acclimatization. In arid areas, woody species like *Populus*, *Prosopis*, *Atriplex*, and *Eucalyptus* species effectively use meager rainfall inputs [168,169]. Plants avoid drainage during dry spells by utilizing water surpluses due to their large storage capacity and deep root development systems. Understanding natural adaptation to salinity and drought in woody plants can accelerate gene integration, producing salt-tolerant and drought-tolerant varieties and maintaining commercial woody species’ productivity [170]. Nevertheless, there are arguments against using and regulating genetically modified wood plants and crops in today’s society. Countries have unique regulations on genome-editing technologies, potentially causing delays or prohibitions in their territories. For instance, the Chinese government has a keen eye for using genetically modified plants. The US Department of Agriculture has no regulations, while the EU requires the some regulation for genetically modified plants [171,172]. Therefore, the commercialization of salt-tolerant and drought-tolerant plants depends on individual laws. Studies on woody plants in salinized soils are crucial for sustainable agro-ecosystem management and genetic information, as native stress-tolerant trees offer valuable biological resources.

## 7. Effect of Water Stress on Hydraulic Traits in *P. euphratica*

Due to their numerous physiological functions, including photosynthesis, transpiration, photoreception, and respiration, leaves are the most important organs for determining the overall growth of a plant [173,174]. The leaves of plants are highly influenced by their environmental resilience [175]. An important aspect of plant physiology and its functional ecology is shown in the connection between leaves and their environment. The way in which leaves develop and evolve could show how the environment has an array of effects on plants, ranging from gene to population [176]. The distribution of water from leaves along the vertical heights of plants is most likely caused by some factors, one of which is likely to be the hydraulic characteristics. As plants grow taller, the leaf-to-atmosphere vapor pressure deficit increases, causing xylem tension and increased gravitational potential [177]. Drought causes plants to lose moisture, which increases the stress in the hydraulic pathways, which can cause cavitation and the development of embolisms (air bubbles) in the water conductivity xylem [143]. This phenomenon creates a blockage of water transport and, when prolonged, may cause hydraulic failure and plant death. To shrink xylem embolisms, which cause plant hydraulic dysfunction, the plant usually improves its water transport capacity by adjusting its water-related functional traits. The *P. euphratica* tree, a dominant species of the desert, is one tree species in the terrestrial ecosystem that develops strong leaves in the face of environmental changes, which subjects it to long-term drought and salt stress tolerance. Many researchers have found that *P. euphratica* has a significant strategy to alleviate the increase in hydraulic constraints along vertical heights [178]. Importantly, poplar saplings exhibit a high degree of phenotypic plasticity in response to water-deficit growing conditions, with reductions in hydraulic stem sensitivity and leaf area being particularly important in postponing the onset of hydraulic failure during an induced drought event. It is interesting to note that *PeGSTU30* increases the hydrophobicity of the active cavity while maintaining strong enzymatic activity [179,180]. Leaf functional characteristics are essential for plants to survive and for ecosystems to function. Understanding how these characteristics coordinate under stress is essential since salinity impacts them. According to Li et al. (2023), *P. euphratica* builds in riparian forests and adjusts and coordinates the functional features of its leaves to survive in saline conditions.

## 8. Conclusions and Future Research Perspectives

In arid areas of China, the fast-growing deciduous tree *P. euphratica* is essential for producing timber. It produces wood and stabilizes sand dunes while tolerating salt, alkalinity, and dryness. This review examines gene family analyses, concentrating on salt and drought tolerance in the Xinjiang and Inner Mongolia sites. Research on *P. euphratica* stress-tolerance genes focuses on its ability to maintain a stable internal water environment under low groundwater levels. New studies are screening functional-coding genes involved in ion transport, antioxidant enzymes, and signal transduction processes. Research suggests that *P. euphratica* can adapt to severe atmospheres by regulating tolerance genes, particularly in leaves related to photosynthesis. Recent applied research, such as the improved tobacco drought tolerance, highlights the importance of gene applications and techniques in enhancing *P. euphratica*’s resilience to drought [2]. It has been demonstrated that genes increase stress tolerance by influencing physiological morphology through the study of the molecular control mechanisms of leaf morphology and physiological alterations in *P. euphratica*. However, the triggers or factors that regulate gene expression in response to stress are controversial among researchers. Some experts believe that chromosomal rearrangements help the tree to adapt, while others argue that environmental stress is the main driver of gene expression. This difference in opinion highlights the need for further investigation and confirmation [181]. Even though various efforts have been made to construct the genome of *P. euphratica* as a model tree plant, there is still a gap in the complete genome for tree breeding. Future studies should focus on this model plant’s identification and the characterization of genes linked to heavy metal pollution under low pH levels. It is important to gain a thorough understanding of the intricate mechanisms underlying *P. euphratica*’s resilience to drought stress and salt stress caused by mycorrhizal connections (a symbiotic relationship between the tree and certain fungi) in desert regions. Furthermore, future studies should employ cutting-edge molecular analysis of gene expression patterns and how they govern mycorrhizal-mediated growth, nutrient uptake, and water uptake in stressful conditions. Future studies should also provide a clear investigation into the molecular mechanisms balancing stress tolerance and development in *P. euphratica.*

## Figures and Tables

**Table 1:** Genome-wide records of gene family in *P. euphratica*.

Species	Identified Genes No.	Gene Family	Functions	Reference
*P. euphratica*	19	HSF	Drought stress	[68]
	22	HSF	Salt stress	[68]
	2 (*PeWRKY45*)	WRKY	Salt stress	[71]
	(*PeWRKY102*)			
	1 (*PebHLH35*)	bHLH	Drought and abscisic acid	[69]
	1 (*PeCPK10*)	CPK	Drought and cold stress	[65]
	1 (*PeSTZ1*)	STZ	Freezing tolerance	[80]
	1 (*PeuGASA15*)	GASA	Drought stress	[74]
	1 (*PeuLAC2*)	LACCASE	Drought stress	[81]
	3	4CL	Salt stress	[97]
	1 (*PeCBL1*; CIPK24,	CBL1	Salt stress	[90]
	CIPK25, and cipk26)			
		AUX-IAA/ARF	Not identified yet	
	1 (*PeHAB1*)	PP2C	Induced by drought and ABA as a negative regulator	[98]
	1 (*PeDREB2*)	DREB2-type	Salt stress	[64]
		AB13-VP1	Not identified yet	
		C2C2-Dof	Not identified yet	
		SBP	Not identified yet	
		MADS	Not identified yet	
		C2C2-GATA	Not identified yet	
		FHA	Not identified yet	
		GARP-G2 like	Not identified yet	
		PeG	Not identified yet	
		C2H2	Not identified yet	
		bZIP	Not identified yet	
		HB	Not identified yet	
		C3H	Not identified yet	
		PHD	Not identified yet	
	*PeSCL7*	GRAS	Salt and drought tolerance	[79]
		AP2-EREBP	Not identified yet	
		MYB-related	Not identified yet	
		SPL	Not identified yet	

## Data Availability

The data that support the findings of this study are available from the corresponding author on reasonable request.
